# Indonesian psychologists’ sensemaking and reported intervention practices in working with women survivors of sexual assault with PTSD symptoms

**DOI:** 10.1186/s40359-026-05093-1

**Published:** 2026-07-14

**Authors:** Ligina Ayudia, Fitri Ariyanti Abidin, Fredrick Dermawan Purba, Annemarie Samuels, Aulia Iskandarsyah

**Affiliations:** 1 Postgraduate Program in Psychology, Faculty of Psychology, Universitas Padjadjaran, Bandung, Indonesia; 2 Department of Psychology, Faculty of Psychology, Universitas Padjadjaran, Bandung, Indonesia; 3 Institute of Cultural Anthropology and Development Sociology, Leiden University, Leiden, the Netherlands; 4 Center for Psychological Innovation and Research, Faculty of Psychology, Universitas Padjadjaran, Bandung, Indonesia; 5 Department of General, Developmental, and Educational Psychology, Faculty of Psychology, Universitas Padjadjaran, Sumedang, Indonesia; 6 Center for Relationship, Family Life and Parenting Studies, Faculty of Psychology, Universitas Padjadjaran, Sumedang, Indonesia

**Keywords:** Sexual assault, Post-traumatic stress disorder (PTSD), Qualitative research, Cultural adaptation, Indonesian psychologist

## Abstract

**Background:**

Sexual assault is a significant public health and human rights concern in Indonesia and is associated with post-traumatic stress disorder (PTSD) symptoms among women survivors. Limited evidence exists on how Indonesian psychologists understand, organise, and adapt psychological interventions for these survivors within complex sociocultural, legal, and institutional contexts. This study explored Indonesian psychologists’ sensemaking and reported intervention practices when working with women survivors of sexual assault who present with PTSD symptoms.

**Method:**

Twenty-one licensed psychologists participated in semi-structured interviews. Data were analyzed using reflexive thematic analysis. The study focused on how interventions were conceptualized, adapted, and delivered in routine practice, including considerations of cultural, familial, legal, and service-system constraints.

**Results:**

Psychologists reported using flexible, modular approaches drawing on CBT-oriented, somatic, humanistic, psychodynamic, problem-solving, and strength-based strategies. Intervention planning emphasized safety, stabilization, therapeutic alliance, and culturally sensitive adaptations, including language, metaphors, family involvement, and religious framing. Clinicians adjusted pacing, session structure, and between-session tasks based on client readiness, stigma, family dynamics, and systemic constraints. Four overarching themes emerged: (1) relationally grounded trauma care, (2) modular and adaptive intervention design, (3) navigation of service and legal constraints, and (4) continuous cultural attunement.

**Conclusions:**

Indonesian psychologists provide trauma-informed care that is relational, adaptive, and culturally situated. These findings may inform training and supervision for psychology trainees and early-career clinicians, while future research should examine survivor perspectives and evaluate intervention outcomes.

**Supplementary Information:**

The online version contains supplementary material available at https://doi.org/10.1186/s40359-026-05093-1.

## Introduction

Women who experience sexual assault often face not only the psychological consequences of trauma, but also stigma, fear of disbelief, family pressure, and institutional barriers when seeking help. In Indonesia, these challenges may shape whether survivors disclose their experiences, access psychological services, and continue care. Understanding how psychological support is provided in this context is therefore important for improving trauma-informed and culturally responsive services. Sexual assault is an unwanted, non-consensual sexual contact that occurs through force, incapacitation, or coercion [1]. Globally, approximately 35.6% of women report having experienced some form of physical or sexual violence, although prevalence estimates vary across regions [2].

In Indonesia, data from the 2016 Survei Pengalaman Hidup Perempuan Nasional (SHPN) indicate that one in three (33.4%) women aged 15–64 years has experienced physical or sexual violence by a partner or non‑partner during her lifetime [3]. More recent administrative data from the Ministry of Women’s Empowerment and Child Protection showed that reported cases of violence against women remained high in 2023 [4]. Sexual assault is closely associated with elevated rates of post‑traumatic stress disorder (PTSD) [1], characterised by re-experiencing (i.e., reliving the event), avoidance, negative thoughts and emotions, and hyperarousal [5]. In Indonesia, survivors’ access to care is also shaped by stigma, victim-blaming, fear of disbelief, and legal or institutional pathways that may affect whether, when, and how they disclose their experiences [6, 7].

A growing body of comparative effectiveness research has examined treatments for PTSD, including pharmacologic and nonpharmacologic interventions [8]. Cognitive interventions focus on how thoughts influence emotions and behaviour (top‑down), helping clients identify distorted memories and maladaptive attitudes, reduce symptoms, and develop more adaptive self‑beliefs and behaviours. Somatic approaches, in contrast, focus on how bodily sensations shape thoughts, emotions, and actions (bottom‑up), fostering awareness of sensations and procedural memories and targeting core dysregulation in the nervous system [9]. By supporting physiological regulation, these approaches aim to reduce debilitating symptoms and unconscious dynamics. Although evidence for somatic approaches is accumulating, further research is needed to establish their efficacy, clarify mechanisms of change, and determine for whom and under what conditions they are most effective. Systematic reviews indicate that trauma‑focused cognitive behavioural therapy (CBT) is effective for many individuals with PTSD symptoms [10], although a substantial proportion of clients do not respond fully or find such interventions difficult to engage with [11]. These clinical considerations suggest that PTSD intervention cannot be understood only as the application of evidence-based techniques; it must also be examined in relation to the social, cultural, legal, and service conditions that shape survivors’ disclosure, engagement, and continuity of care [12, 13]. These conditions are particularly important in sexual assault cases because stigma, victim-blaming, and fear of disbelief may shape whether survivors disclose the assault, seek help, or continue psychological care. Thus, silence and taboo are not simply background cultural features; they are part of the context in which intervention access and engagement occur.

Given the high incidence of sexual assault, understanding how psychological interventions are implemented in different cultural, health-system, and structural contexts, particularly in Asian countries, is crucial. Many interventions that have demonstrated efficacy in reducing PTSD symptoms were originally developed and tested in Western settings. Their direct adoption may not fully account for the culturally, geographically, politically, and economically situated nature of care in non‑Western societies such as Indonesia. Adapting interventions to local values, family structures, and religious beliefs is therefore essential for clinical relevance and effectiveness, but cultural adaptation alone may be insufficient [14, 15]. Implementation also depends on how people access care, what services are available, health-system constraints, economic conditions, and structural factors that shape the marginalisation of women and survivors of sexual assault [13, 16] Framing barriers to care solely as matters of “culture” risks obscuring broader global systems of inequality and colonial legacies of power that continue to influence whose knowledge, interventions, and service models are treated as normative [17, 18]. From an implementation science perspective, the effectiveness of psychological interventions depends not only on intervention content, but also on the context in which interventions are delivered, adapted, and sustained [12, 16]. However, limited research has examined how psychologists in Indonesia actually organise, deliver, and adapt PTSD-related psychological interventions for women survivors of sexual assault in routine practice. This gap is important because clinicians must navigate not only therapeutic techniques, but also survivors’ readiness, family and religious expectations, stigma, legal processes, institutional constraints, and limited service resources.

This study focused on psychologists rather than survivors because the aim was to examine how psychological interventions are conceptualised, organised, and adapted by professionals who deliver care within Indonesian clinical, protection-service, and institutional settings. Psychologists’ accounts can illuminate how intervention decisions are shaped by referral pathways, legal processes, service constraints, professional training, and cultural expectations. However, their perspectives cannot substitute for survivors’ lived experiences of receiving care. Survivor-centred research remains essential for evaluating whether these approaches are experienced as helpful, acceptable, or harmful.

### Purpose and research questions

Given this gap, this study aimed to explore how Indonesian psychologists describe the practical organisation, delivery, and adaptation of psychological interventions when working with women survivors of sexual assault who present with PTSD symptoms. Rather than evaluating treatment outcomes, this study examines what psychologists report doing in routine practice, how they make decisions about intervention content and session structure, and how they adapt care to cultural, legal, relational, and service-system constraints. This focus is consistent with implementation science perspectives, which emphasise that intervention delivery and effectiveness are shaped by context, including organisational, social, and service-system conditions [12, 13]. The research questions were designed to address this gap by examining four connected dimensions of practice: the intervention approaches psychologists use, the ways sessions are structured, the challenges that shape implementation, and the cultural adaptations made in Indonesian service contexts.

The study addressed the following research questions:

i) What types of psychological interventions are employed by Indonesian psychologists to support women survivors of sexual assault who present with PTSD symptoms?
ii) How are intervention sessions structured, both intra‑session and inter‑session, to support symptom relief and cognitive–emotional processing?
iii) What challenges do psychologists encounter when implementing these interventions, particularly with respect to emotional regulation, narrative disclosure, and social stigma?
iv) In what ways are interventions culturally adapted to reflect Indonesian values and the broader legal, relational, and service contexts in which care is delivered?

## Method

The author(s) declared that financial support was received for this work and/or its publication. This study was supported by the Padjadjaran University Research Grant (No. 1549/UN6.3.1/PT.00/2023). Publication support was funded by Universitas Padjadjaran through the Indonesian Endowment Fund for Education (LPDP) on behalf of the Indonesian Ministry of Higher Education, Science and Technology and managed under the EQUITY Program (Contract No. 4303/B3/DT.03.08/2025 and 3927/UN6.RKT/HK.07.00/2025). The funding bodies had no role in the study design, data collection, analysis, interpretation, or reporting of the study. No additional external funding was received for this study.

### Research design

A qualitative phenomenological-hermeneutic approach was used within an interpretivist–constructivist paradigm. This design was selected because the study aimed to understand psychologists’ situated meanings, clinical decision-making, and interpretations of intervention practice rather than to measure treatment outcomes or test predefined hypotheses. The phenomenological component supported exploration of psychologists’ lived experiences of providing care, while the hermeneutic component supported interpretation of how those experiences were shaped by cultural, legal, institutional, and service-system contexts. This design enables in-depth understanding of intervention processes, session structures, and the meanings psychologists attach to their clinical decisions within cultural, legal, institutional, and service-system contexts. This orientation was selected because the study aimed to understand situated clinical meanings and practices, rather than to measure intervention effectiveness or test predetermined hypotheses. Specifically, we sought to understand how psychologists make sense of intervention choices, client engagement, barriers to treatment, session organisation, and cultural adaptation in routine practice. The phenomenological component enabled exploration of psychologists’ lived experiences of providing care, while the hermeneutic component supported interpretation of how these experiences were shaped by cultural, legal, institutional, and service-system contexts.

### Reporting checklist

This manuscript was prepared in accordance with the Consolidated Criteria for Reporting Qualitative Research (COREQ), a 32-item checklist for reporting qualitative studies using interviews and focus groups. The checklist was used to guide reporting of the research team and reflexivity, study design, participant recruitment, data collection, data analysis, and presentation of findings. The COREQ checklist was used to guide transparent reporting of the research team, reflexivity, recruitment, data collection, analysis, and presentation of findings. It did not determine the study design; rather, it supported complete reporting of the qualitative procedures used in the study.

### Philosophical stance and qualitative orientation

This study was conducted within an interpretivist–constructivist paradigm, which assumes that realities are multiple, context‑dependent, and shaped through social interaction. In this view, Indonesian psychologists’ accounts of their work with women survivors of sexual assault are treated as situated interpretations rather than objective descriptions of fixed clinical facts. Knowledge is understood as co‑constructed between researchers and participants through dialogue, and attention is given to how professional roles, institutional contexts, and sociocultural norms inform these meanings.

A ‘Big Q’ qualitative orientation guided the study, with an emphasis on in‑depth, contextualised understanding rather than measurement, prediction, or hypothesis testing. The phenomenological–hermeneutic design was chosen to explore psychologists’ lived experiences of providing care and to interpret how they make sense of intervention processes, session structures, and cultural adaptations in a collectivist, resource‑limited setting. This philosophical stance underpins the use of semi‑structured interviews and reflexive discussions incorporated throughout the analytic process. In practice, this meant that interviews were designed to invite psychologists’ interpretations of their clinical decisions rather than to verify whether they followed predefined treatment protocols. Follow-up questions were used to explore how participants made sense of client readiness, stigma, family involvement, legal processes, and institutional constraints. During analysis, the research team treated participants’ accounts as situated professional narratives and examined how meanings were shaped by service setting, cultural expectations, and the researchers’ own clinical assumptions.

### Ethical considerations

The study was approved by the Research Ethics Committee of Universitas Padjadjaran (No. 1357/UN6.KEP/EC/2023). All procedures involving human participants were performed in accordance with the relevant ethical guidelines and regulations, including the principles of the Declaration of Helsinki. All participants were licensed psychologists who received written and verbal information about the study aims, procedures, potential risks and benefits, and their rights, including the right to withdraw at any time without consequences, before providing written informed consent for participation, audio‑recording, and anonymized use of their data. Because the interviews focused on psychologists’ work with women survivors of sexual assault and post‑traumatic stress, participants were reminded that they could pause or stop the interview whenever they wished. All names and identifying details of clients, colleagues, institutions, and locations mentioned by participants were replaced with pseudonyms during transcription. Audio files and transcripts were stored on encrypted, password-protected drives accessible only to the core research team, and a de-identified dataset was used for analysis.

### Participants and sampling

Participants **(***N* = 21**)** were selected purposively based on inclusion criteria which required them to be licensed psychologists and members of the Indonesian Association of Psychologists (Himpunan Psikologi Indonesia, HIMPSI). Licensed psychologists were selected because they are formally authorised to conduct psychological assessment and provide psychological intervention in Indonesian clinical and protection-service settings. The study focused on this professional group to obtain a detailed account of how intervention approaches are selected, explained, structured, and adapted by practitioners who routinely work with sexual assault-related trauma. The purpose was not to determine whether these interventions improved survivor outcomes, but to understand the clinical reasoning, service constraints, and cultural considerations shaping intervention delivery. This focus provides a foundation for subsequent research that should include survivor perspectives and outcome evaluation.

Eligibility was confirmed through documented experience in handling cases of sexual assault and trauma, validated via the provision of a professional licence. A multi-pronged recruitment strategy was adopted, drawing from professional networks, institutional contacts, and services affiliated with the Protection of Women and Children service organizations (Perlindungan Perempuan dan Anak, PPA), as well as psychological bureaus, clinics, and university psychology service units. The exact number of psychologists who declined or did not respond was not systematically recorded. No participants withdrew after providing consent.

Participants included 18 women and 3 men, aged 28–50 years, who voluntarily agreed to take part in the study. Most participants had been practising for two to five years. They worked across a range of service settings: 43% in government-owned Women and Children Protection Units (PPAs), 37% in NGOs focused on violence against women, and the remainder in hospitals, psychological bureaus, private clinics, or university psychology service units. All participants held at least a professional psychology degree. Data saturation was assessed iteratively during data collection and preliminary analysis and was considered achieved when no substantially new codes or patterns emerged from additional interviews, particularly regarding intervention approaches, session structure, implementation barriers, and cultural adaptation. As a token of appreciation, participants received a gift voucher and an honorarium totaling IDR 25,000, approximately USD 1.60–1.70 at the time of the study. This was intended as a symbolic token of appreciation rather than compensation for professional time.

### Data collection

Data were collected using semi-structured interviews, which were audio-recorded in Bahasa Indonesia with participant consent and subsequently transcribed verbatim. All interviews were conducted by the first author. Participants were informed that the study aimed to understand psychologists’ experiences in delivering psychological interventions for women survivors of sexual assault who present with PTSD symptoms. Prior to each interview, participants completed a sociodemographic questionnaire covering age, professional experience, affiliation, and location. The interview protocol was explained, and participants provided consent to audio-recording in Bahasa Indonesia. The semi-structured interview guide was reviewed by the research team before data collection. Interviews included an initial section focused on understanding qualities needed to handle sexual trauma cases, types of assault encountered, and challenges in the field; followed by a main interview addressing practical treatment approaches, session durations, client complaints, and obstacles in therapy. The interview guide explored psychologists’ experiences of working with women survivors of sexual assault, including the types of cases encountered, referral pathways, initial assessment, therapeutic goals, intervention approaches, session structure, between-session tasks, treatment completion, follow-up, perceived barriers to engagement, cultural adaptation, family involvement, religious and gendered considerations, legal or institutional constraints, and training or supervision needs. Follow-up questions were used to explore how participants made decisions about pacing, client readiness, disclosure, emotional regulation, and the balance between therapeutic and medicolegal roles.

Interviews were conducted either in person at the participant’s workplace or online via Google Meet, depending on the participant’s preference. No non-participants were present during the interviews. No repeat interviews were conducted. Interview duration ranged from 45 to 110 min, with an average length of 60 min. A total of 21 audio recordings were obtained. Field notes were made during and after interviews to record contextual observations and initial analytic reflections. Transcripts were not returned to participants for correction.

Selected quotations were translated from Bahasa Indonesia into English for publication. To support consistency of meaning, translated quotations were checked by bilingual members of the research team against the original transcripts, with attention to idiomatic expressions, emotional tone, and culturally specific meanings.

### Data analysis

Data were analysed using the six phases of thematic analysis as outlined by Braun and Clarke (2006) [19]. First, the 21 audio-recorded interviews were transcribed, and the research team familiarised themselves with the content by reading the transcripts and clarifying unclear parts by re-listening to the recordings. Initial patterns and potential codes were noted. Next, NVivo 12 software was used to develop an inductive codebook, with the first author and an independent coder coding the transcripts separately. Coding differences were discussed to refine code definitions, explore alternative interpretations, and strengthen analytic consistency rather than to calculate inter-rater reliability. The final codebook was then agreed upon by the coders. The initial codes were grouped into sub-categories and categories, which were then examined for potential themes. In line with Braun and Clarke’s approach to thematic analysis, themes were developed by examining repeated patterns of meaning across participants’ accounts, rather than by counting the frequency of codes alone [19]. Codes were compared within and across transcripts to identify how psychologists described intervention delivery, client engagement, barriers, session structure, and cultural adaptation. Candidate themes were then reviewed against the coded extracts and the full dataset to assess whether they captured coherent and distinct aspects of the phenomenon [19]. A coding tree was developed to document the relationship between codes, sub-categories, categories, and themes. Participant quotations were selected to illustrate each theme and sub-theme and were identified using participant codes and service settings, such as NGO, private bureau, hospital, or government-owned practice, to preserve anonymity while retaining contextual information.

These themes were refined into one contextual section and four analytic themes: building therapeutic safety and relational trust; assembling modular intervention content; structuring care within service and legal constraints; and adapting care to cultural, familial, religious, and gendered contexts. Each theme was divided into sub-themes, while irrelevant codes were discarded. Subthemes were used to describe variation within each major analytic area, including barriers to participation, reasons for ending treatment, behavioural and cognitive between-session tasks, initial and regular session attendance, treatment completion, follow-up, language sensitivity, familial values, societal and religious norms, and collectivist preferences. Where relevant, differences across service settings, such as NGOs, private bureaus, hospitals, and government-owned practices, were noted to show how implementation varied by context. The coherence and fit of the themes were reviewed against the coded extracts and full dataset before the final thematic structure was confirmed. Findings were checked against coded extracts and the full dataset to ensure consistency between participants’ accounts, analytic claims, and selected quotations. Emerging interpretations were shared informally with a small subset of psychologist participants and peer clinicians to assess resonance with clinical experience, but formal member checking was not conducted. The analysis concluded with a report detailing the themes, supported by relevant quotations from the interviews to illustrate the findings.

### Rigor and trustworthiness

This study addressed trustworthiness using four criteria: credibility, transferability, dependability, and confirmability [20]. Credibility was supported through prolonged engagement with the data, repeated reading of transcripts, and independent coding by both the first author and an external qualitative researcher. Coding discrepancies and differing interpretations were resolved through collaborative discussion, leading to a consensus codebook and thematic structure. Emerging interpretations were shared informally with a small subset of psychologist participants and peer clinicians; this informal feedback was used to assess whether the themes resonated with broader clinical experience but did not constitute formal member checking.

Transferability was facilitated by providing thick description of the settings (including NGOs, hospitals, and government agencies), participants’ backgrounds, and the relevant sociocultural context, enabling readers to judge the applicability of findings elsewhere. Dependability was strengthened by maintaining a clear audit trail documenting analytical and methodological decisions throughout the NVivo-assisted analysis. Confirmability was enhanced through regular team debriefings, allowing examination of how researchers’ professional backgrounds and values influenced data interpretation. These combined strategies aimed to produce a transparent, interpretable account grounded in participants’ narratives and context.

### Researcher positionality and reflexivity

The research team consisted of clinical psychologists, a clinical anthropologist, and mental health researchers with training in trauma‑focused interventions and qualitative methods in Indonesia. The first author is a clinician–researcher, doctoral candidate, and clinical psychologist. At the time of the study, the first author was affiliated with the Faculty of Psychology, Universitas Padjadjaran. The interviewer was female. The first author had previous professional contact with some institutions involved in services for women and children affected by violence, but did not have a therapeutic relationship with participants. The first author has collaborated with NGOs and government agencies providing services for women and children affected by violence, which facilitated access to practicing psychologists and helped build collegial rapport during interviews. Participants were informed about the researcher’s academic role, clinical background, and interest in understanding psychologists’ experiences of delivering interventions for sexual assault survivors in Indonesian service contexts.

At the same time, this proximity to the field may have fostered assumptions that formal psychological services and evidence‑based interventions are intrinsically beneficial. To address this, the team used reflexive discussions during analysis to question whether emerging themes overemphasised clinical technique at the expense of structural constraints, legal pressures, and survivors’ social realities. For example, when early coding focused heavily on intervention methods, the team revisited transcripts to examine how psychologists also described service limitations, stigma, family expectations, and medicolegal responsibilities. This led to greater analytic attention to implementation context and cultural adaptation rather than treating intervention delivery as a purely technical process.

## Results

The findings are organised into one contextual section followed by four analytic themes. The contextual section briefly describes the forms of sexual assault and survivor difficulties reported by psychologists. The four analytic themes then explain how psychologists made sense of trauma care in practice: (1) building therapeutic safety and relational trust; (2) assembling modular intervention content; (3) structuring care within service and legal constraints; and (4) adapting care to cultural, familial, religious, and gendered contexts. This structure was used to move beyond procedural categories such as intra-session work, inter-session tasks, and session attendance, and instead highlight broader patterns of meaning in how psychologists implemented psychological care in routine Indonesian service settings.

**Fig. 1:**
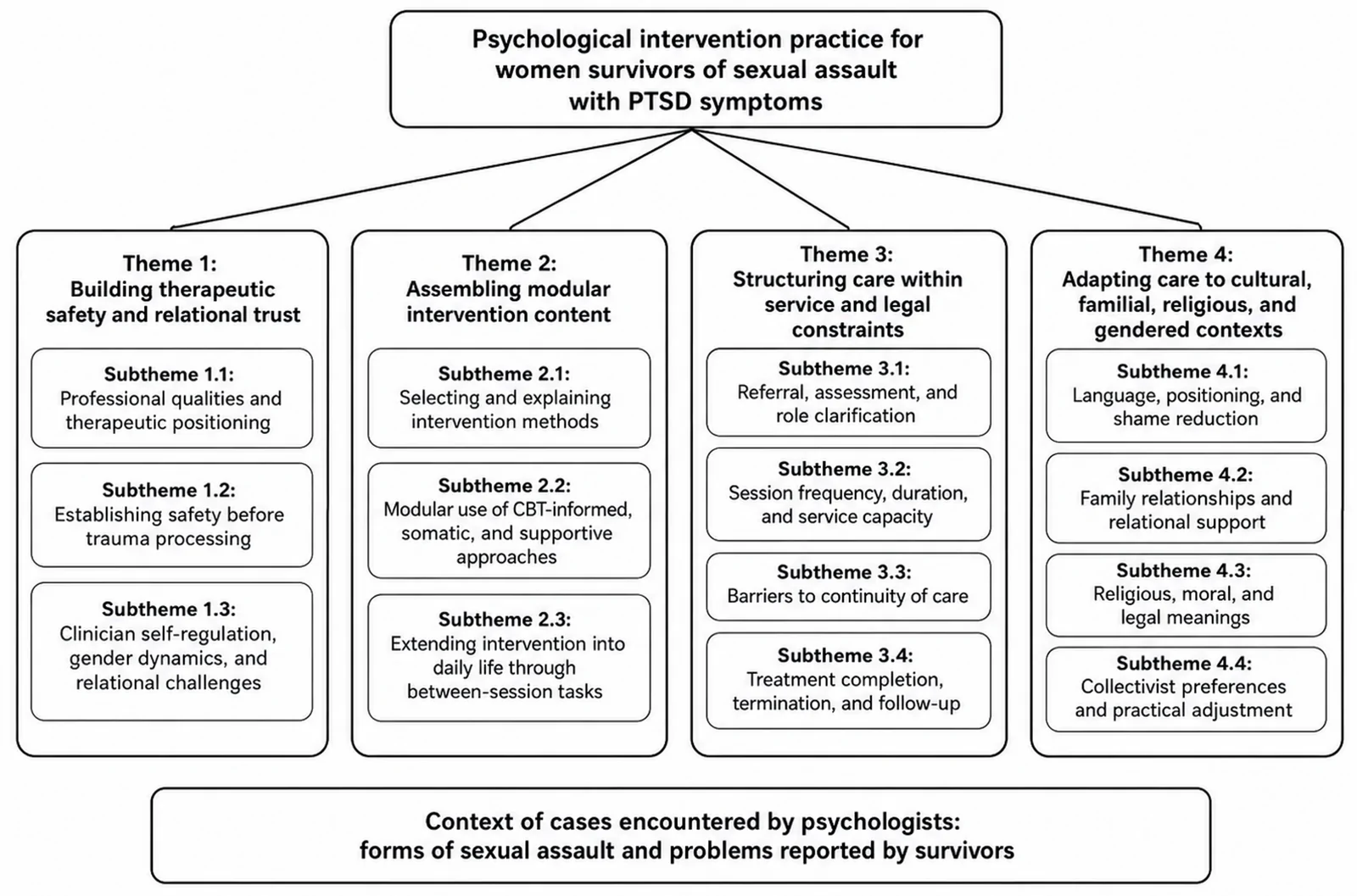
Overview of themes and subthemes in psychological intervention practice. Note. Contextual information situates the four analytic themes but was not treated as a separate theme

Figure 1 presents an overview of the four analytic themes and their subthemes, showing how psychologists described trauma care as relationally grounded, modular, structurally constrained, and culturally adapted.

### Contextual background to the reported intervention practices

Before presenting the analytic themes, we briefly summarise the forms of sexual assault and survivor difficulties that psychologists reported encountering in practice. This information is included only as contextual background to situate the intervention processes described below. As the study draws on clinician interviews rather than survivor interviews, this section is not presented as a direct account of survivors’ lived experiences and is not treated as a separate analytic theme. The analytic focus remains on psychologists’ sensemaking, intervention decisions, and adaptation of care.

Participants reported working with women survivors who had experienced various forms of sexual assault, including unwanted sexual contact, coercive sexual acts, rape, and sexual violence occurring within dating, family, institutional, or community contexts. Psychologists also described a range of trauma-related difficulties among survivors, such as fear, shame, self-blame, intrusive memories, avoidance, emotional dysregulation, difficulties in trusting others, social withdrawal, and disruption in daily functioning.

These contextual descriptions help explain the clinical situations in which psychologists made intervention decisions. Participants emphasised that intervention planning required careful consideration of survivors’ readiness, emotional safety, disclosure process, family responses, cultural expectations, and legal or institutional constraints. The following themes therefore examine how psychologists made sense of these complex situations and how they selected, structured, and adapted interventions for women survivors of sexual assault with PTSD symptoms.

#### Forms of sexual assault

Across their caseloads, psychologists encountered a wide spectrum of sexual violence. Survivors reported verbal harassment such as catcalling, various forms of physical violence including trafficking, dating and domestic violence, forced abortion, rape, fornication, and sexual abuse of children, as well as non‑physical violations such as marriage coercion and extortion. Participants also described cases involving information and communications technology (ICT)‑based violence, especially the non‑consensual distribution of intimate images or ‘revenge porn’. Physical abuse, particularly rape, was the most frequently reported form of violence, and most women who experienced sexual assault were adults aged 18 years or older.

The client population seeking psychological support covered a broad age range, from early childhood to late adulthood. Many survivors accessed services through private bureaus, clinics, or hospitals, where interventions could be tailored to their specific needs. Government‑owned services such as PPAs played a key role, though clients were often referred from community organisations, health professionals, or social services rather than self‑referring directly.

#### Problems reported by survivors

Psychologists described survivor difficulties across interconnected cognitive, emotional, physical, and behavioural domains. Cognitively, many clients struggled with intrusive memories and flashbacks, persistent rumination, concentration problems, and repetitive negative thoughts, including self-blame, shame, guilt, confusion, feelings of worthlessness, and suicidal ideation. Several survivors also reported anticipatory fear and difficulties recounting events in a coherent chronological sequence.

Emotionally, participants frequently presented with unstable affect, anxiety, fear, sadness, numbness, depressive mood, and loss of interest. Overt expressions such as freezing, mutism, crying spells, and withdrawal from social interactions were commonly observed, alongside more covert struggles involving self-blame, shame, and negative self-perceptions.

Across accounts, somatic complaints were described as one of the most salient ways in which trauma-related distress became visible in clinical encounters. Rather than appearing as isolated physical symptoms, bodily pain, autonomic arousal, sleep disturbance, and sensory discomfort were frequently discussed alongside anxiety, flashbacks, hypervigilance, avoidance, and difficulties with trust and intimacy. This pattern led psychologists to understand survivors’ distress as embodied as well as cognitive and emotional, shaping their emphasis on grounding, stabilisation, and careful pacing before deeper trauma processing.

Behaviourally, many survivors showed patterns of avoidance, freezing or withdrawal, sleep and eating disturbances, self-isolation, repetitive behaviours, excessive engagement in work as a coping mechanism, and tendencies toward self-harm. Psychologists therefore described survivor difficulties as involving intrusive memories, negative mood and cognitions, avoidance, hyperarousal, and somatic distress across age groups and types of assault.

### Theme 1: building therapeutic safety and relational trust

Psychologists described therapeutic work with survivors of sexual assault as beginning with the creation of safety, trust, and emotional stability. This theme captures the relational and professional foundations that participants viewed as necessary before more structured trauma-focused work could occur. Rather than treating rapport as a preliminary step only, psychologists described alliance-building, self-regulation, ethical positioning, and sensitivity to gendered dynamics as ongoing conditions for therapeutic engagement.

#### Subtheme 1.1: professional qualities and therapeutic positioning

Participants described a combination of professional competencies and personal qualities they considered essential when working with sexual assault survivors. Core competencies included administering and interpreting psychological assessments, applying forensic skills, and understanding relevant legal frameworks. Alongside these technical skills, psychologists emphasised personal traits such as emotional awareness, flexibility, humility, self-regulation, sensitivity, and a sustained willingness to keep learning. These qualities were viewed as crucial for establishing trust and a strong therapeutic alliance between psychologists and survivor-clients, particularly when addressing highly sensitive trauma histories.

#### Subtheme 1.2: establishing safety before trauma processing

Building rapport was described as an active, ongoing process. Psychologists supported self‑regulation and emotional stabilisation, asked about clients’ daily lives, invited questions, fostered a sense of safety and comfort, introduced enjoyable or manageable tasks, clarified goals and ethical boundaries, explored biographical histories, and used empathic listening and storytelling to encourage disclosure.

For example, when describing self‑regulation strategies, one psychologist noted: “Usually it’s towards emotional regulation because that’s what they need most to be able to calm down,” while another emphasised, “Relaxation, grounding for example, if the client is in a tense condition” (HN, SR, NGOs). These accounts illustrate a broader pattern in which psychologists prioritised stabilisation before trauma narration or cognitive restructuring. Emotional regulation was not described as a discrete technique only, but as a prerequisite for safety, disclosure, and subsequent therapeutic work. Cognitive reflection and restructuring were also used to help clients identify strengths and reframe traumatic experiences: “What kind of reflection does she have? What strengths does she have that make her feel in control” (HN, NGO). Establishing “positioning” early in the process was important, particularly in institutional settings, to clarify the psychologist’s role and differentiate therapeutic work from legal procedures. As one psychologist explained, they needed to introduce their professional status and the institution’s protective function so that victims, who might have already told their story to police, could feel safer sharing their experiences in a different context (LT, government‑owned practice).

#### Subtheme 1.3: clinician self-regulation, gender dynamics, and relational challenges

On the provider side, psychologists described multiple challenges, including familiarity effects when working with clients they already knew, high case complexity, difficulties engaging children, limited forensic expertise, constraints associated with online service delivery, insufficient sexual and reproductive health knowledge, gender dynamics that affected rapport-building, perceived safety, and clients’ willingness to disclose traumatic experiences, personal biases, and difficulties with self-regulation. Some practitioners also reported challenges in establishing and maintaining therapeutic relationships in cases involving sexual violence, particularly when survivors felt ashamed, feared being judged or blamed, struggled to trust the psychologist, or found it difficult to disclose traumatic experiences. Practitioners described needing to build safety gradually, avoid pushing clients to recount details before they were ready, and maintain clear boundaries while showing empathy and validation.

These challenges collectively highlighted participants’ emphasis on the need for structured, ethically grounded, and culturally resonant clinical competencies when working with survivors of sexual violence. Participants underscored the importance of clear ethical boundaries, systematic assessment skills, and a solid grounding in legal and forensic frameworks to navigate complex cases while minimizing harm and secondary victimization. At the same time, culturally resonant competencies particularly gender sensitivity, awareness of stigma and shame, and attunement to relational norms were viewed as essential for fostering safety and trust within the therapeutic relationship.

Across accounts, clinicians’ capacity for self-regulation emerged as a critical professional requirement, particularly in the context of repeated exposure to traumatic narratives. As one psychologist noted, *“There is need for self-control*,* because the stories of these clients tend to be disturbing”* (KB, private hospital). This underscores the ethical necessity of clinician self-awareness and emotional regulation to sustain therapeutic presence and prevent burnout or countertransference.

Gender-related dynamics further reinforced the need for culturally informed practice. One male participant explained, *“I find it most challenging*,* one of which is that when the client is female compared to male*,* the approach is different”* (MA, private bureau), pointing to the importance of gender-sensitive frameworks in assessment and intervention. Such accounts suggest that without culturally resonant competencies, clinicians may struggle to adapt their therapeutic stance to the relational and sociocultural meanings embedded in sexual violence experiences.

### Theme 2: assembling modular intervention content

Participants rarely described applying a single manualised treatment from beginning to end. Instead, they assembled modular components from CBT-informed, somatic, humanistic, psychodynamic, problem-solving, and strength-based approaches. Intervention content was selected according to survivors’ readiness, emotional regulation capacity, service time limits, and the perceived risk of overwhelming clients. This theme therefore brings together both in-session techniques and between-session tasks as connected elements of flexible trauma care.

#### Subtheme 2.1: selecting and explaining intervention methods

Intervention methods were selected based on the client’s condition, the anticipated length and intensity of treatment (brief versus more comprehensive work), and organisational constraints. Psychologists described providing concise explanations of the techniques they planned to use, including expected processes and potential effects. For example, one clinician who frequently used brainspotting stated: “Well, I usually introduce the intended therapy, it is called brainspotting. A brief explanation is offered initially, followed by what is expected of the clients, and the impacts” (NA, government‑owned practice).

#### Subtheme 2.2: modular use of CBT-informed, somatic, and supportive approaches

Across PPAs, government bureaus, and hospitals, counselling was often the default modality because few psychologists had formal certification in specific therapeutic modalities, such as CBT, brainspotting, Somatic Experiencing, or Theraplay. Only two of the twelve psychologists in these settings reported holding such modality-specific certifications, which restricted the range of interventions that could be offered. Commonly used approaches included cognitive‑behavioural, humanistic, psychoanalytic, and strength‑based methods, supplemented by brainspotting, Somatic Experiencing, and Theraplay. CBT‑informed counselling was most frequently applied, as it helped reduce distress and modify negative perceptions. At the same time, participants were acutely aware of its limitations. Several highlighted that CBT could feel confrontational and might trigger defensive responses, making it hard for survivors to tell their stories or regulate intense emotions. In this study, certification refers to formal training or certification in specific therapeutic modalities, including CBT, brainspotting, Somatic Experiencing, and Theraplay, rather than certification as a psychotherapist in a general or psychodynamic sense.

One psychologist commented, “I used both MBCT and CBT, which was quite effective for clients, specifically those suffering from symptoms of trauma” (SR, NGO). Others described adapting their approach when clients were not yet ready for cognitive work: “I counselled clients using a CBT method when the problem is related to mindset… But this method appears ineffective if the clients are defensive or not ready to change a particular mindset. I try to ensure the clients are strong by reducing emotional pressures, through the adoption of relaxation methods… then after which CBT is applied” (AP, NGO). Another clinician noted that challenging cognitive distortions around disgust and shame following rape was often difficult in a context of strong societal stigma (SR, NGO). Starting CBT too early could inhibit storytelling: “The application of CBT at the initial stage makes it impossible to tell the story” (SR, government‑owned practice). Participants also reflected that CBT’s emphasis on examining the “truth” of thoughts might be challenging for particularly sensitive clients, or for those whose educational background made abstract cognitive work more difficult (F, FP, NGOs).

#### Subtheme 2.3: extending intervention into daily life through between-session tasks

Inter-session work referred to tasks that clients completed between sessions to consolidate therapeutic gains and apply new skills in daily life. Psychologists described using both behavioural and cognitive assignments to foster independence and support generalisation of learning.

Behavioural tasks included exercises to strengthen adaptive coping, maintain reproductive and physical health, develop social skills, practice self‑protection, and encourage self‑exploration. One psychologist described encouraging clients to stay engaged in meaningful activities: “Doing activities, keep clients busy with activities to prevent sad thoughts caused by the trauma, which has already been stored in long-term memory (LTM), which affects the limbic system and others” (TS, private bureau). Here, the participant appeared to be referring to traumatic memories that had become encoded in long-term memory and continued to influence emotional and physiological responses. Another emphasised building social competence and help‑seeking: “Most of what I teach is how the victim can be independent, know which people to ask for help when the need arises, including the assessment. That is what I initially teach the victims” (A, NGO).

Cognitive assignments focused on cognitive reconstruction, expressive writing, emotional stabilisation, self-compassion, and self-awareness. One psychologist highlighted the ambivalence of asking clients to attend to bodily sensations: “Paying attention to the breathing process, and body sensations can be triggering. This was because when the clients experienced assault the best way not to feel the trauma was to remain numb, except triggered, which could be traumatizing” (AS, private bureau). Another described gradually cultivating emotional awareness as a foundation for cognitive restructuring: “I usually teach the victim how to recognise and identify specific emotions. Therefore, when it has to do with cognitive identification for restructuring, we are comfortable, because the victims are already aware of the triggers, causes, and other factors” (FP, NGO).

### Theme 3: structuring care within service and legal constraints

Psychologists described intervention structure as shaped not only by clinical judgement, but also by referral pathways, legal processes, organisational capacity, and survivors’ socioeconomic circumstances. Decisions about assessment, number of sessions, treatment pacing, termination, and follow-up were therefore negotiated within service constraints. This theme brings together material previously described under session attendance, treatment completion, and barriers to participation, in order to show how care structure was shaped by institutional and medicolegal realities.

#### Subtheme 3.1: referral, assessment, and role clarification

Initial sessions most often took place after a referral from family members, social services, community organizations, or the police in the aftermath of a sexual assault. Younger clients (under 17 years) were typically brought by parents or community actors, whereas women aged 17 years and above sought help for more varied reasons, including both legal processes and personal concerns about ongoing distress. One psychologist working in a government‑owned Women and Child Protection institution explained that walk‑in cases were rare: “Because this is a Women and Child Protection institution, it is rare for people to visit directly, victims are usually referred. This means that the story had already been heard, in addition, the referrer could be from other institutions, specifically from the police station” (L, works in government‑owned practice).

Another psychologist described how some cases were linked directly to court proceedings, while others reflected survivors’ own efforts to seek emotional support: “This usually depends on several cases, some maybe investigated in court, where the victim gets a referral for psychological examination, which becomes a form of evidence. The victim can also be treated of emotional and psychological problems, although there are also those who seek help personally. The victims who usually seek help on their own view sexual activities as scary, despite being of reproductive age and should already be married. These individuals seek help, because it is needed even when married, and are also concerned. Therefore, there are two types of clients, some are referred for legal processes, and those who are private, mainly interested in what happened, and the bothering impact” (VP, works in NGO).

A recurring structural challenge was the overlap between therapeutic and medicolegal roles. Psychologists working in government-owned and protection-service settings often met survivors after police, legal, or referral processes had already begun. This shaped how psychologists introduced their role, clarified confidentiality and boundaries, and distinguished therapeutic care from forensic or evidentiary procedures. Participants described this role clarification as important because survivors may have already repeated their stories to police or other institutions before meeting the psychologist.

During these first encounters, psychologists conducted comprehensive assessments using interviews, observation, and, when indicated, psychological tests. Interviews were used to obtain detailed, first‑hand accounts of the assault and its consequences, forming the basis for tailored intervention plans. Observations of behaviour, affect, and physiological responses helped clinicians gauge safety, emotional regulation, and early indicators of alliance. In PPAs and NGOs, assessment could be supplemented by objective measures such as Culture Fair Intelligence Test (CFIT), Advanced Progressive Matrices (APM), or Figure Reasoning Test (FRT), and projective techniques such as the Baum Test (BAUM) or Draw-a-Person (DAP). One psychologist highlighted the importance of early cognitive assessment: “The assessment sessions two and three are usually the most important. This intelligence assessment requires the use of simple tests such as CFIT or APM. The aim was to understand the survivor’s cognitive functioning” (NA, works in government‑owned practice). In this context, intelligence testing was described as a way of tailoring communication, determining whether additional support was needed during assessment, and ensuring that intervention plans were developmentally and cognitively appropriate, rather than as an evaluation of the survivor’s credibility or worth.

#### Subtheme 3.2: session frequency, duration, and service capacity

Participants emphasised that the number and duration of sessions varied according to intervention type, service constraints, and client circumstances, and that these factors strongly influenced adherence. Counselling typically ranged from 3 to 12 sessions, psychotherapy from 5 to 10 sessions, and hypnotherapy from 3 to 4 sessions, with individual sessions usually lasting 1 to 1.5 h. One psychologist working in a government‑owned unit described a relatively structured sequence: “For example, the four to eight sessions depended on the client, start from initial intake, assessment, followed by therapy in sessions four, five, six, and seven. During session eight evaluation was carried out, and it was the longest. This was because in the Regional Technical Implementation Unit for Women and Children Protection (Unit Pelaksana Teknis Daerah Perlindungan Perempuan dan Anak, UPTD PPA), the sessions are usually brief due to the many other clients who need to be treated” (NA, works in government‑owned practice).

Others described briefer courses of treatment or the need to refer on for more intensive work. As one NGO‑based psychologist noted, “Incidentally, my clients are probably easier to handle, and these individuals tend to get better in three or four sessions. Some take longer time but are finally referred due to the need psychotherapy, and hypnotherapy” (A, works in NGO). Another explained that in PPA settings, survivors were often seen only two or three times, which made it necessary to prioritize what could be addressed in a short time frame (F, works in NGO). Service availability and socioeconomic conditions further limited continuity: “It depended on the condition, which could be ideally more than 20. However, when in the field with few clients, characterized by middle income earners, low education tends to be infrequent due to interference with working hours … This was because the sessions clashed with the working hours, and UPDT PPA were not open at night or holiday hours. Some may be transferred to work elsewhere, if, the case is charged to court” (VL, works in government‑owned practice).

Within these constraints, psychologists aimed to maintain a consistent session length. One clinician stated, “In cases of violence, ideally, the maximum duration was 1 to 1.5 hours if possible” (F, works in NGO). Another linked session duration to both administrative realities and a somatic understanding of trauma: “1 to 1.5 hours is what I give. The first reason is administrative, the second focuses on the trauma of renegotiation, which needs to be brief. Based on SE classes, trauma refers to anything that is too fast, intense, and absolutely not good for the body” (AS, works in a private bureau).

Psychologists generally agreed a fixed rhythm for sessions at the outset of therapy and then adjusted it in response to client progress or symptom change. For some, this meant setting an upper limit on how often clients could be seen: “I generally attend to clients always and at most once every two weeks” (ER, works in NGO). Others described starting with weekly meetings and then spacing sessions out as clients stabilized, or bringing treatment to a close when goals had been met: “This was usually done once a week at the beginning, for example, the progress of the victims determines the duration, including the need to reduce the timing, or terminate the session” (NI, works in NGO).

#### Subtheme 3.3: barriers to treatment participation

Psychologists identified barriers to treatment participation at both the client and clinician levels. On the client side, they described overwhelming emotions, fear of legal processes, limited financial and practical resources, work–schedule constraints, and belief systems influenced by stigma and shame. Lack of resources and constraining beliefs were the most frequently reported obstacles. As one psychologist noted, “Because they have a middle income, usually because of work conflicts they can’t continue” (VL, government-owned practice), referring to how work commitments and service opening hours restricted attendance. Another emphasised the influence of stigma: “There are many factors, firstly we also have a stigma in society, so clients are embarrassed or afraid to tell their story” (DK, government-owned practice).

Psychologists also reported additional contextual barriers, including lack of family or community support, denial of survivors’ experiences, and the psychological strain associated with legal or forensic procedures. These circumstances were described as contributing to disengagement and high dropout rates, with survivors often withdrawing due to shame, distrust, avoidance, or dissociative reactions.

Finally, across interviews, limited institutional support and persistent stigma experienced by clients were identified as additional barriers to consistent treatment participation. These external constraints, combined with clinicians’ internal challenges, further emphasize the necessity of structured and ethically grounded competencies that are responsive to cultural, gendered, and contextual realities in the provision of psychological care for survivors of sexual violence.

#### Subtheme 3.4: treatment completion, termination, and follow-up

Treatment completion and termination were shaped by both clinical improvement and practical constraints. Psychologists described ending or pausing treatment for practical reasons, including educational and employment demands, general busyness, court processes, and socioeconomic constraints. Clinically informed reasons included clients feeling better, not yet being ready to process trauma, or feeling uncomfortable continuing. Socioeconomic pressures and clients’ perception that they had improved were reported as the most common reasons for ending sessions.

One psychologist working in a government setting explained that middle‑income clients often faced scheduling clashes because “the UPDT PPA is usually not open at night or during holidays” (VL, government‑owned practice). In some cases, legal processes effectively displaced therapy: “Because the case was immediately taken to court, there was no need for therapy” (NA, government‑owned practice).

Clinicians also described ending or pausing treatment when clients did not yet feel ready to engage deeply with traumatic material: readiness was monitored session by session, sometimes using brief evaluation scales used to ask clients how they felt after each meeting (SR, NGO). Psychologists frequently made termination decisions collaboratively with clients and, where possible, used short‑term monitoring to ensure stability before fully ending therapy.

Criteria for treatment completion combined standardized measures with clinical judgement. Psychologists sometimes used tools such as the BDI and HPQ to monitor changes in symptoms and functioning. However, treatment was usually considered complete based on a broader constellation of improvements, including adaptive coping skills, achievement of collaboratively agreed goals, improved personal and social functioning, subjective improvements in well‑being, symptom reduction, integration of psychoeducation, perceptual shifts, and the ability to identify and challenge negative thoughts. One psychologist described relying on questionnaire scores while also recognizing their limitations: “If the initial complaints are reduced, the results of the HPQ tend to decrease. But this is challenging due to difficulty in distinguishing whether the complaint is real or not” (VL, works in government‑owned practice).

Subjective and relational criteria were therefore at least as important as symptom measures. Another clinician explained using a brief monitoring period after apparent improvement: “Then, after that, evaluation was carried out for another two weeks. If no relapse was observed within the 2‑week period … then the clients are in good condition. Therefore, the victims can go back to respective daily activities, such as school, and work again. The entire session is immediately terminated. But if after the two‑week evaluation, the symptoms reappear, then the therapy session need to be reconducted” (NA, works in PPA). Across accounts, treatment completion was primarily grounded in subjective assessments of personal and social functioning, supported but not determined by symptom measures.

Follow-up practices varied considerably between psychologists and service settings. Some clinicians routinely offered follow-up contacts after termination, particularly when survivors had experienced severe violence but had adequate support systems. As one NGO-based psychologist described: “Even when one session had been completed, because maybe the individual is a victim of violence, but has a good support system, and well informed, I will still follow up for two weeks to monitor the person” (ER, works in NGO). Others scheduled planned check-ins after one or two months, while some noted that post-termination monitoring was often difficult to provide because of resource constraints. In contrast, psychologists in private practice reported that clients rarely returned once therapy had ended unless new problems emerged. Together, these accounts suggest that follow-up care was valued as part of safe termination, but was unevenly implemented because of service capacity, client circumstances, and organisational norms.

### Theme 4: adapting care to cultural, familial, religious, and gendered contexts

Cultural adaptation was not described as a discrete technique or final step in treatment. Rather, psychologists portrayed it as an ongoing clinical stance that shaped language, therapeutic positioning, family involvement, religious framing, pacing, and responses to gendered expectations. This theme captures how participants adapted care to survivors’ relational worlds while also navigating stigma, moral judgement, and the legal meanings attached to sexual violence in Indonesia.

#### Subtheme 4.1: language, positioning, and shame reduction

Participants emphasised the importance of using non‑stigmatizing, culturally attuned language that fits the client’s social context. Rather than framing clients as “problematic” or “at fault”, psychologists sought to affirm their status as victims and to position them within supportive narratives. One clinician described this succinctly as “positioning the client as a victim and then adapting to the social situation” (DK, works in NGO). Such linguistic choices were viewed as essential for reducing shame and facilitating disclosure in a context where sexual assault is often moralised.

#### Subtheme 4.2: family relationships and relational support

Family dynamics were central to how psychologists understood and worked with survivors. Narratives were frequently contextualized in ways that acknowledged the importance of family roles and responsibilities, while gently addressing patterns that contributed to distress. Therapists explored how parents and other relatives responded to the assault, and how these responses shaped the survivor’s emotions. As one psychologist explained, “I always ask ‘how do you feel?’ especially regarding your relationship with your parents…” (LS, work in NGO). By foregrounding family relationships in this way, clinicians aimed to reduce shame, strengthen support, and align interventions with clients’ relational worlds.

#### Subtheme 4.3: religious, moral, and legal meanings

Religious, moral, and legal meanings were often intertwined in how psychologists framed safety, rights, responsibility, and healing. Participants described drawing on religious language or practices when appropriate, while also recognising that legal processes and social norms could intensify shame or fear of disclosure. This required psychologists to balance culturally resonant forms of support with careful attention to survivors’ autonomy and protection.

Social and religious norms were woven into therapeutic work through gender‑sensitive facilitation, optional spiritual practices, and coping strategies that resonated with local moral frameworks, such as patience and acceptance. Psychologists also stressed the need to understand relevant laws and religious–cultural expectations when working with children and adults. One participant noted that clinicians “must know the child protection laws and also understand the cultural and religious context in Indonesia” (VL, works in government‑owned practice). Integrating these dimensions helped therapists frame safety, rights, and healing in ways that were both legally informed and culturally meaningful.

#### Subtheme 4.4: collectivist preferences and practical adjustment

Intervention methods were further adjusted to accommodate collectivist preferences, including the value placed on togetherness, modest self‑disclosure, and socially appropriate pacing. Psychologists described using small, comfortable groups or dyadic formats, scheduling sessions around clients’ daily routines, and reinforcing change in ways that fit community expectations. One clinician described an intentionally non‑directive start: “At first I would just listen to whatever he wanted to tell me…” (LS, working in NGO), highlighting the priority given to creating a safe relational space before introducing more structured techniques. Another commented on calibrating the number of sessions to what felt feasible and acceptable: “Yes, the shortest course of treatment is around four sessions… the longest is six to twelve sessions” (DK, working in NGO). Together, these adaptations illustrate how therapists balanced clinical aims with practical and cultural considerations in a collectivist setting.

## Discussion

This qualitative study examined how Indonesian psychologists working with women survivors of sexual assault who present with PTSD symptoms describe their intervention strategies, session structures, implementation challenges, and cultural adaptations. These findings suggest that, within a context of high stigma and constrained resources, clinicians draw eclectically on cognitive, somatic, and supportive approaches while engaging in continuous cultural attunement to family, gender, and religious norms, rather than implementing single, protocolised models of care.

The main contribution of this study is not evidence of treatment effectiveness, because clinical outcomes were not evaluated. Rather, the study provides a contextually grounded account of how Indonesian psychologists select, adapt, and implement interventions, and these findings may also be particularly useful for psychology trainees, early-career psychologists, and supervisors as guidance for trauma-informed practice and supervision. Therefore, references to perceived usefulness or improvement should be understood as clinicians’ perspectives, not as evidence that particular interventions reduced PTSD symptoms. Future research should examine survivors’ experiences and evaluate outcomes such as symptoms, functioning, safety, and quality of life.

Conceptually, this study extends the literature by showing that culturally responsive trauma care in Indonesia is not only a matter of adapting intervention content, but also of negotiating therapeutic roles, moral meanings, family expectations, legal processes, and service constraints throughout the intervention process. These patterns suggest that trauma care in this setting is best understood not as the delivery of a fixed intervention package, but as a process of clinical negotiation. Psychologists had to balance evidence-informed techniques with survivors’ readiness, stigma, family expectations, legal processes, institutional demands, and limited service capacity. This reveals a form of trauma care that is modular, relational, and context-dependent.

The findings suggest that Indonesian psychologists rarely applied PTSD interventions as complete manualised protocols. Instead, they assembled modular elements from CBT-oriented, somatic, humanistic, psychodynamic, problem-solving, and strength-based approaches in response to client readiness, service time limits, legal involvement, and the risk of overwhelming survivors. This pattern both resembles global PTSD intervention models that emphasise psychoeducation, cognitive restructuring, narrative processing, and arousal management, and extends this literature by showing how such components are pragmatically reorganised within resource-constrained Indonesian service settings. This interpretation is consistent with evidence supporting trauma-focused CBT across diverse populations [21, 22], while also reflecting implementation challenges commonly reported in low- and middle-income mental health systems [23].

Somatic components including breathing exercises, body scanning, Somatic Experiencing, and brainspotting were used to regulate arousal and improve survivors’ tolerance for bodily sensations. Compared with more established cognitive and exposure-based PTSD interventions, these somatic practices appeared to function primarily as stabilisation tools that helped survivors manage bodily arousal before engaging in narrative or cognitive work. This finding is important because it shows how Indonesian psychologists integrated body-oriented strategies pragmatically within routine care, even when the evidence base for some somatic modalities remains less developed than that for trauma-focused CBT. These practices align with psychophysiological and cross-cultural perspectives models that conceptualize PTSD as involving disruptions in autonomic regulation as well as cognitive meaning-makin [24]. Consistent with cross-cultural adaptation research, clinicians noted that intensive cognitive exposure early in treatment was often overwhelming, necessitating a stabilization-first approach before deeper trauma processing could occur [25].

Session flow followed phase-based trauma principles prioritising safety, alliance building, and early regulation skills. Clinicians typically began by normalising trauma responses, clarifying roles, and introducing grounding techniques before progressing to narrative exploration or cognitive reappraisal consistent with international psychotrauma guidelines that discourage rapid exposure when clients remain dysregulated [25].

Between-session practices included behavioural activation tasks (resuming daily routines, increasing self-protective behaviours), social support mobilisation, and cognitive assignments such as journaling or self-compassion exercises. These strategies are consistent with behavioural and narrative interventions shown to improve functioning and reduce distress in humanitarian and LMIC settings [26] Psychologists emphasised tailoring homework to survivors’ socioeconomic realities, reflecting earlier findings that poorly adapted tasks may exacerbate burden rather than enhance empowerment.

Barriers experienced by clinicians mirrored patterns observed in cross-cultural PTSD research. Client-level obstacles shame, fear of blame, concerns about disclosure, financial hardship, and apprehension about legal involvement, align with studies documenting barriers to help-seeking among underserved survivors of sexual assault [27]. Provider-level challenges included emotional fatigue, limited specialised training, gendered power dynamics, and personal biases, consistent with literature on the demands placed on practitioners working with trauma and sexual violence. Systemic constraints high caseloads, restricted service hours, expectations for dual medicolegal and therapeutic roles reflect broader implementation barriers in LMIC mental health systems [23, 28]. Overall, the findings underscore that the success of CBT, somatic, or integrative techniques cannot be separated from the structural and relational contexts in which care is delivered.

A key conceptual contribution of this study is that cultural adaptation appeared not as a discrete procedural step, but as an ongoing clinical stance. Psychologists adapted language, pacing, family involvement, religious framing, and therapeutic positioning throughout the intervention process. This suggests that, in Indonesian trauma care, cultural adaptation is embedded in everyday clinical judgement and relational work rather than added after selecting an intervention model. Psychologists adapted language to avoid terms associated with “madness” or moral failing, reframed survivors as victims rather than violators of social expectations, and used culturally resonant metaphors to validate trauma reactions. Using cultural adaptation frameworks, these practices can be understood as involving both surface-level adaptations, such as language, metaphors, and communication style, and deeper structural adaptations, such as attention to family roles, religious meanings, gendered stigma, and the moral and legal positioning of sexual violence. These adaptations align with frameworks emphasising modification of both surface and deep-structure elements while preserving core therapeutic mechanisms [29].

Family involvement was encouraged when it enhanced safety or reduced isolation, resonating with research on the centrality of family systems in collectivist cultures. Clinicians also incorporated religious framing such as concepts of acceptance, divine compassion, or spiritual grounding when appropriate, without compromising psychological principles of change. At the same time, therapists navigated tensions between cultural expectations of female modesty, widespread victim-blaming, and survivors’ needs for autonomy, emotional expression, and validation. This dynamic negotiation reflects findings from culturally adapted CBT and broader cross-cultural intervention research showing that adaptation is an iterative, relational process shaped by clinician judgment, client context, and sociocultural norms [30, 31].

Collectively, these findings suggest that cultural adaptation in Indonesian trauma practice is not a discrete procedural step but an ongoing clinical stance responsive to survivors’ lived realities. Such flexibility may be particularly important in LMIC settings, where strict adherence to manualised protocols may be insufficient without contextual grounding.

These findings also have implications for how evidence-based trauma interventions are conceptualised in settings where services are fragmented across protection, health, legal, and community systems. Rather than assuming that intervention fidelity and cultural adaptation are opposing priorities, the present findings suggest that fidelity to trauma-informed principles may require flexible adaptation to survivors’ readiness, safety, family context, and service realities. In this sense, culturally responsive trauma care in Indonesia involves preserving core principles of safety, stabilisation, validation, and empowerment while adapting delivery format, language, pacing, and relational involvement to local contexts.

## Strengths & limitations

This research is, to our knowledge, the first qualitative study to provide an in-depth overview of the strategies and frameworks adopted by Indonesian psychologists working with women survivors of sexual assault who present with PTSD symptoms. Nevertheless, several limitations should be noted. First, the sample was relatively small and included few male psychologists, which may limit the diversity of professional perspectives represented and the transferability of the findings to other regions or service settings in Indonesia. Second, all interviews were conducted in Bahasa Indonesia and only selected excerpts were translated into English for publication. Although a careful translation process was used and the research team attended to idioms, emotional tone, and cultural references, some nuances of meaning were inevitably lost or softened in translation. Third, in order to protect confidentiality, potentially identifying contextual details and culturally specific examples were occasionally modified or omitted; this may constrain how fully the affective and cultural weight of certain narratives can be conveyed. Fourth, the study examined psychologists’ accounts rather than survivors’ lived experiences of receiving care. Therefore, the findings reflect clinician perspectives on intervention processes and should not be interpreted as evidence of how survivors themselves experience, evaluate, or benefit from these interventions. Future research should include survivor perspectives to examine which aspects of care are experienced as helpful, harmful, culturally acceptable, or difficult to access.

## Conclusion

This study provides one of the first in‑depth qualitative accounts of how Indonesian psychologists working with women survivors of sexual assault who present with PTSD symptoms design and deliver psychological interventions. The findings show that clinicians draw flexibly on CBT‑oriented, humanistic, psychodynamic, problem‑solving, strength‑based, and somatic strategies while prioritizing safety, emotional regulation, and a strong therapeutic alliance in the face of time pressure, role conflict, and high levels of stigma. Rather than applying manualized treatments in a uniform way, psychologists assemble brief, modular interventions and continuously adjust their methods to clients’ readiness, family dynamics, and service constraints.

The results highlight that cultural adaptation is not a separate step but an ongoing clinical stance in which language, metaphors, family involvement, and religious framing are negotiated to validate survivors’ experiences without reinforcing harmful norms. This culturally attuned, phase‑based practice is broadly consistent with international evidence on trauma‑focused interventions yet also illustrates how their feasibility and impact depend on structural conditions within Indonesian mental health and protection systems. The findings may be particularly useful for psychology trainees, early-career psychologists, and supervisors who require contextually grounded guidance on how trauma-informed care is organised in practice. These findings highlight practical strategies that can inform training, supervision, and early-career clinical development. However, these findings should not be interpreted as evidence that the interventions reduce PTSD symptoms. Future research should include survivors’ perspectives and evaluate outcomes such as symptoms, functioning, safety, and quality of life.

These findings suggest three practical priorities. First, psychologist training should include phase-based trauma care, trauma-focused CBT components, somatic stabilisation skills, risk assessment, and gender-sensitive communication for sexual assault cases. Second, services should provide regular supervision and peer consultation to help clinicians manage vicarious trauma, countertransference, role conflict, and ethically complex medicolegal cases. Third, policy and service systems should improve continuity of care by extending service hours, clarifying referral pathways between PPAs, NGOs, hospitals, police, and courts, and developing guidance on confidentiality, family involvement, and the boundary between therapeutic and forensic roles.

## Implication for practice, policy, and future research

From a clinical perspective, the findings highlight the need for phase‑based, culturally responsive trauma care in Indonesian services for sexual assault survivors. Psychologists in this study already prioritized safety, stabilization, and alliance‑building, and flexibly integrated CBT‑oriented, somatic, and supportive strategies; targeted training could further strengthen competence in trauma‑focused CBT components, somatic stabilization techniques, and risk management in cases that involve ongoing legal processes or family conflict. These findings may also inform training programs and supervision for psychology trainees and early-career clinicians, providing guidance on culturally responsive trauma-informed practice. Regular supervision and peer support structures are also needed to address vicarious trauma and emotional fatigue among clinicians working with highly stigmatized forms of violence. These recommendations are based on descriptive accounts and should not be interpreted as evidence of treatment effectiveness.

At the policy and system levels, the results point to the importance of expanding access to trauma‑informed services beyond urban centers, extending opening hours to accommodate working survivors, and clarifying the boundaries between therapeutic and medicolegal roles in Women and Children Protection Units. Professional associations and regulatory bodies could use these data to develop practice guidelines that integrate global evidence with Indonesian legal, religious, and cultural frameworks, including clear standards for confidentiality, family involvement, and collaboration with police and social services. Partnerships between NGOs, hospitals, and government agencies may help ensure continuity of care when survivors move between systems.

Future research should build on this study by incorporating survivors’ perspectives on what aspects of therapy feel most and least helpful, and by examining how gender, socioeconomic status, ethnicity, and region shape both access to care and experiences of intervention. Participatory and innovative qualitative approaches, such as community-based participatory research and online photovoice, may be useful for centring survivors’ perspectives and generating more grounded recommendations for trauma-informed services [32, 33]. Mixed‑methods and longitudinal designs could explore how specific combinations of cognitive, somatic, and culturally adapted strategies relate to changes in PTSD symptoms, functioning, and quality of life over time. Future studies should incorporate survivors’ perspectives to evaluate which interventions are most helpful and assess clinical outcomes. Such work would provide an empirical basis for refining modular, culturally grounded intervention models that are feasible in low‑resource Indonesian settings and potentially transferable to other low‑ and middle‑income countries with similar sociocultural dynamics.

## Supplementary Information

Supplementary Material 1.

## Data Availability

The datasets generated and/or analyzed during the current study are available from the corresponding author on reasonable request. No supplementary data are available.
