# Supplementary material for: Indonesian psychologists’ sensemaking and reported intervention practices in working with women survivors of sexual assault with PTSD symptoms
Source: BMC Psychol. 2026 Jul 14;14:1604. doi: 10.1186/s40359-026-05093-1 (PMC13741713; doi:10.1186/s40359-026-05093-1)
Supplement: Supplementary file 1 [file 40359_2026_5093_MOESM1_ESM.docx]

Consolidated criteria for reporting qualitative studies (COREQ): 32-item checklist

**Developed from:**

Tong A, Sainsbury P, Craig J. Consolidated criteria for reporting qualitative research (COREQ): a 32-item checklist for interviews and focus groups. *International Journal for Quality in Health Care*. 2007. Volume 19, Number 6: pp. 349 – 357

**MANUSCRIPT TITLE:**

Psychological intervention process and structure in women survivors of sexual assault with PTSD symptoms: qualitative research among Indonesian psychologists

| **No. Item** | **Guide questions/description** | **Reported on Page #** |
| --- | --- | --- |
| **Domain 1: Research team and reflexivity** |  |  |
| *Personal Characteristics* |  |  |
| 1. Inter viewer/facilitator | Which author/s conducted the interview or focus group?  The interviews were conducted by the first author. | Section 2.6 Data Collection |
| 2. Credentials | What were the researcher’s credentials? (E.g. PhD, MD)  The research team consisted of clinical psychologists, a clinical anthropologist, and mental health researchers. The first author was a clinician–researcher, doctoral candidate, and clinical psychologist. | Section 2.9 Researcher positionality and reflexivity |
| 3. Occupation | What was their occupation at the time of the study?  The first author was a clinician–researcher, doctoral candidate, and clinical psychologist affiliated with the Faculty of Psychology, Universitas Padjadjaran at the time of the study. | Section 2.9 |
| 4. Gender | Was the researcher male or female?  The interviewer was female | Section 2.9 |
| 5. Experience and training | What experience or training did the researcher have?  The research team had training in trauma-focused interventions and qualitative methods in Indonesia. The first author had collaborated with NGOs and government agencies providing services for women and children affected by violence. | Section 2.9 |
| *Relationship with participants* |  |  |
| 6. Relationship established | *Was a relationship established prior to study commencement?*  The first author had previous professional contact with some institutions involved in services for women and children affected by violence, but did not have a therapeutic relationship with participants. This institutional familiarity facilitated access and collegial rapport. | Section 2.9 |
| 7. Participant knowledge of the interviewer | *What did the participants know about the researcher? (e.g. personal goals, reasons for doing the research).*  Participants were informed about the researcher’s academic role, clinical background, and interest in understanding psychologists’ experiences of delivering interventions for sexual assault survivors in Indonesian service contexts. | Sections 2.6 and 2.9 |
| 8. Interviewer characteristics | *What characteristics were reported about the interviewer/facilitator? (e.g. Bias, assumptions, reasons and interests in the research topic)*  The manuscript reports that the first author’s proximity to the field may have shaped assumptions that formal psychological services and evidence-based interventions are beneficial. Reflexive discussions were used to examine whether early interpretations overemphasised clinical techniques at the expense of structural constraints, legal pressures, and survivors’ social realities. | Section 2.9 |
| **Domain 2: Study design** |  |  |
| *Theoretical framework* |  |  |
| 9. Methodological orientation and Theory | *What methodological orientation was stated to underpin the study? (e.g. grounded theory, discourse analysis, ethnography, phenomenology, content analysis).*  The study used an interpretivist–constructivist paradigm, a Big Q qualitative orientation, and a phenomenological–hermeneutic design to explore psychologists’ lived experiences and interpret how these experiences were shaped by cultural, legal, institutional, and service-system contexts. | Sections 2.1 and 2.3 |

| *Participant selection* |  |  |
| --- | --- | --- |
| 10. Sampling | *How were participants selected? (e.g. purposive, convenience, consecutive, snowball)*  Participants were selected purposively. Inclusion criteria required participants to be licensed psychologists, members of the Indonesian Association of Psychologists (HIMPSI), and experienced in handling sexual assault and trauma cases. | Section 2.5 Participants and Sampling |
| 11. Method of approach | *How were participants approached? (e.g. face- to-face, telephone, mail, email)*  Participants were recruited through professional networks, institutional contacts, services affiliated with Protection of Women and Children organisations, psychological bureaus, clinics, and university psychology service units. | Section 2.5 |
| 12. Sample size | *How many participants were in the study?*  The study included 21 licensed psychologists. | Section 2.5 |
| 13. Non-participation | *How many people refused to participate or dropped out? Reasons?*  The exact number of psychologists who declined or did not respond was not systematically recorded. No participants withdrew after providing consent. | Section 2.5 |
| *Setting* |  |  |
| 14. Setting of data collection | *Where was the data collected? (e.g. home, clinic, workplace)*  Interviews were conducted either in person at participants’ workplaces or online via Google Meet, depending on participant preference. | Section 2.6 |
| 15. Presence of non- participants | *Was anyone else present besides the participants and researchers?*  No non-participants were present during the interviews. | Section 2.6 |
| 16. Description of sample | *What are the important characteristics of the sample? (e.g. demographic data, date)*  Participants included 18 women and 3 men aged 28–50 years. Most had been practising for two to five years. They worked across government-owned Women and Children Protection Units, NGOs, hospitals, psychological bureaus, private clinics, and university psychology service units. | Section 2.5 |
| *Data collection* |  |  |
| 17. Interview guide | *Were questions, prompts, guides provided by the authors? Was it pilot tested?*  A semi-structured interview guide was used. Interviews covered qualities needed to handle sexual trauma cases, types of assault encountered, field challenges, treatment approaches, session duration, client complaints, and obstacles in therapy. The guide was reviewed by the research team before data collection. | Section 2.6 |
| 18. Repeat interviews | *Were repeat interviews carried out? If yes, how many?*  No repeat interviews were conducted | Section 2.6 |
| 19. Audio/visual recording | *Did the research use audio or visual recording to collect the data?*  Interviews were audio-recorded in Bahasa Indonesia with participant consent. | Section 2.6 |
| 20. Field notes | *Were field notes made during and/or after the interview or focus group?*  Field notes were made during and after interviews to record contextual observations and initial analytic reflections. | Section 2.6 |
| 21. Duration | *What was the duration of the inter views or focus group?*  Interviews lasted 45–110 minutes, with an average duration of 60 minutes. | Section 2.6 |
| 22. Data saturation | Was data saturation discussed? Data saturation was assessed iteratively during data collection and preliminary analysis. It was considered achieved when no substantially new codes or patterns emerged, particularly regarding intervention approaches, session structure, implementation barriers, and cultural adaptation. | Section 2.5 |
| 23. Transcripts returned | *Were transcripts returned to participants for comment and/or correction?*  Transcripts were not returned to participants for correction | Section 2.6 |
| **Domain 3: analysis and findings** |  |  |
| *Data analysis* |  |  |
| 24. Number of data coders | How many data coders coded the data?  Two coders were involved: the first author and an independent coder. | Section 2.7 Data analysis |

| 25. Description of the coding tree | *Did authors provide a description of the coding tree?*  A coding tree was developed to document the relationship between codes, sub-categories, categories, and themes. | Section 2.7 |
| --- | --- | --- |
| 26. Derivation of themes | *Were themes identified in advance or derived from the data?*  Themes were derived inductively from the data. Codes were compared within and across transcripts, grouped into sub-categories and categories, and refined into one contextual section and four analytic themes. Themes were developed by examining repeated patterns of meaning rather than by counting code frequency alone. | Section 2.7 |
| 27. Software | *What software, if applicable, was used to manage the data?*  NVivo 12 was used to manage and organise the data. | Abstract and Section 2.7 |
| 28. Participant checking | *Did participants provide feedback on the findings?*  Emerging interpretations were shared informally with a small subset of psychologist participants and peer clinicians to assess resonance with clinical experience, but formal member checking was not conducted. | Sections 2.7 and 2.8 |
| *Reporting* |  |  |
| 29. Quotations presented | *Were participant quotations presented to illustrate the themes/findings? Was each*  *quotation identified? (e.g. participant number)*  Participant quotations were presented to illustrate themes and subthemes. Quotations were identified using participant codes and service settings, such as NGO, private bureau, hospital, or government-owned practice, to preserve anonymity while retaining contextual information. | Sections 2.7 and 3 Results |
| 30. Data and findings consistent | *Was there consistency between the data presented and the findings?*  Findings were checked against coded extracts and the full dataset to ensure consistency between participants’ accounts, analytic claims, and selected quotations. Results sections include analytic summaries supported by participant quotations. | Sections 2.7 and 3 Results |
| 31. Clarity of major themes | *Were major themes clearly presented in the findings?*  Major themes are clearly presented in the Results as four analytic themes: building therapeutic safety and relational trust; assembling modular intervention content; structuring care within service and legal constraints; and adapting care to cultural, familial, religious, and gendered contexts. | Section 3 Results |
| 32. Clarity of minor themes | Is there a description of diverse cases or discussion of minor themes?  Subthemes are described under each major theme, including professional qualities, safety before trauma processing, clinician self-regulation and gender dynamics, modular intervention content, between-session tasks, referral and assessment, session frequency, barriers to participation, treatment completion, and cultural adaptation through language, family, religion, legal meanings, and collectivist preferences. | Section 3 Results |
